# Changes in Registered Nurse Employment Plans and Workplace Assessments

**DOI:** 10.1001/jamanetworkopen.2024.21680

**Published:** 2024-07-18

**Authors:** Christopher R. Friese, Barbara R. Medvec, Deanna J. Marriott, Lara Khadr, Marissa Rurka Wade, Melissa Riba, Marita G. Titler

**Affiliations:** 1 Center for Improving Patient and Population Health, University of Michigan, Ann Arbor; 2 Department of Systems, Populations, and Leadership, School of Nursing, University of Michigan, Ann Arbor; 3 Applied Biostatistics Laboratory, School of Nursing, University of Michigan, Ann Arbor; 4 Center for Health and Research Transformation, Ann Arbor, Michigan

## Abstract

**Importance:**

The US registered nurse (RN) workforce is in flux, with high rates of burnout, intention to leave, and vacancies. Rapid, repeated assessments of the nursing workforce can help hospital executives and policymakers enact effective recruitment and retention strategies.

**Objective:**

To identify changes in practicing RNs’ employment plans and workplace assessments between the 2022 and 2023 surveys.

**Design, Setting, and Participants:**

This survey study compared data collected from the Michigan Nurses’ Study at 2 time points: February 22 to March 1, 2022, and May 17 to June 1, 2023. Practicing RNs with an active, unrestricted license in Michigan and a valid individual email address were included.

**Main Outcome and Measures:**

The primary outcome was nurses’ intention to leave their current position within 1 year. In the 2023 survey, nurses who planned to leave were queried on their next career step and the primary reason for their planned departure. Workplace assessments included questions about abusive or violent workplace events, emotional exhaustion, job satisfaction, the practice environment’s delivery of high-quality care, and the clinical setting’s safety rating. Regression analysis was used to examine workplace assessments and personal factors associated with planned departures.

**Results:**

This study obtained data on 9150 nurses (6495 females [71.0%]) and 7059 nurses (5134 females [72.7%]) responding to the 2022 (response rate, 8.3%) and 2023 (response rate, 7.4%) surveys, respectively. In the 2023 survey, 32.0% (2259) of nurses planned to leave their position, compared with 39.1% (3576) in the 2022 survey. Of these nurses, 957 (41.8%) planned to leave their current employer but remain in nursing, with workloads as the most frequently cited reason (29.4% [672]). Compared with the 2022 cohort, nurses in the 2023 sample reported less workplace abuse or violence (4591 [50.2%] vs 3063 [43.4%]; *P* < .001), fewer understaffed shifts (4407 [48.2%] vs 2898 [41.0%]; *P* < .001), and less frequent use of mandatory overtime (1709 [18.7%] vs 824 [11.7%]; *P* < .001). Factors associated with increased likelihood for planned departures included workplace abuse or violence (odds ratio [OR], 1.39; 95% CI, 1.05-1.82) and higher emotional exhaustion scores (OR, 3.05; 95% CI, 2.38-3.91). Favorable practice environments (OR, 0.37; 95% CI, 0.22-0.62) and excellent clinical setting safety ratings (OR, 0.28; 95% CI, 0.14-0.56) were associated with lower likelihood of planned departure.

**Conclusions and Relevance:**

Results of this study showed that nurses reported improved workplace conditions in the 2023 vs the 2022 survey; however, planned departure rates, abusive or violent events, and unsafe conditions remained high, and understaffing remained a primary concern for most nurses. Health system leaders and policymakers should prioritize initiatives that support nurse retention and reduce potential workforce instability.

## Key Points

**Question:**

How have nurses’ workplace assessments and intention to leave their workplace changed from the 2022 to the 2023 Michigan Nurses’ Study?

**Findings:**

In this survey study of 9150 and 7059 nurse participants in the 2022 and 2023 surveys, respectively, significantly fewer nurses were planning to leave their workplace in 2023 than in 2022 (32.0% vs 39.1%). Workplace assessments improved in the 2023 survey; however, planned departure rates, abusive or violent events, and unsafe conditions remained high, and understaffing remained a primary concern.

**Meaning:**

Findings of this study suggest that improved working conditions are likely to promote nurse retention; health system leaders and policymakers should prioritize initiatives that support nurse retention and reduce potential workforce instability.

## Teaser

This survey study compares career plans as well as associated workplace and personal factors as self-reported by practicing registered nurses in the 2022 and 2023 Michigan Nurses’ Study.

## Introduction

Registered nurse (RN) workforce challenges predate the COVID-19 pandemic.^1^ Multiple studies have identified high levels of burnout and intention to leave their positions among nurses.^2^ Reports vary on future nursing workforce adequacy. A 2017 report estimated a shortage of 510 000 nurses by 2030.^3^ A 2024 analysis projected that by 2035, nurse employment would rebound close to prepandemic estimates.^4^

In the early phases of the COVID-19 pandemic, high proportions of health care workers reported their intention to leave their positions, especially female workers and nurses.^5^ News reports suggested that nurses were leaving their positions, citing unsafe conditions.^6^ Hospital leaders reported acute vacancies and demand for expensive temporary personnel, triggering congressional inquiries.^7^ Nurses reported increased vacancies, citing the workload as a primary reason.^1^ Yet the size and trajectory of the problem are unclear, which has hindered policy implementation.

To narrow knowledge gaps, a team at the University of Michigan launched the Michigan Nurses’ Study in 2022.^8^ Using a statewide mailing list, the team queried RNs on their employment plans and workplace assessments. The survey found that 39% of respondents in active practice planned to leave their employer within the year. Factors associated with departures included younger age, abusive events, emotional exhaustion, unfavorable workplaces, and staffing and resource inadequacy.^8^ The 2022 cohort was not queried on their future plans or the reason for their planned departure. It is important to discern whether nurses are leaving their employers (a retention issue) or the nursing profession (an overall supply issue).

We repeated this survey in 2023 to identify changes in practicing RNs’ employment plans and workplace assessments from the 2022 survey. We added questions to the 2023 survey to explore reasons for departures and factors associated with the departure plans. The data generated can inform recruitment and retention strategies locally as well as workforce policy proposals nationally.

## Methods

### Survey Procedures

Survey methods have been previously reported and were identical in both survey years.^8^ The State of Michigan’s publicly available database of RNs was used to identify potential participants; 99% of all nurses in Michigan provide an email address during licensure renewal. Up to 3 email invitations with a survey link were sent 8 days apart via Qualtrics (Silver Lake). No incentives were offered, but individuals could request copies of study findings. The 2022 survey was open from February 22 to March 1, 2022, and the 2023 survey was open from May 17 to June 1, 2023. The University of Michigan Institutional Review Board deemed the study exempt from review. All participants provided online informed consent. This survey study followed the Strengthening the Reporting of Observational Studies in Epidemiology (STROBE)^9^ and the American Association for Public Opinion Research (AAPOR) reporting guidelines.

Survey eligibility included an active, unrestricted license in Michigan and a valid individual email address. Individuals were excluded if the Michigan database indicated a disciplinary process or license suspension and if their email address was not formatted correctly or the email could not be delivered. We restricted the present analysis to those who stated on the survey they were in active clinical practice.

### Measures

Variable selection was informed by the National Academy of Medicine framework^10^ and prior work.^8^ All data were obtained from survey respondents. Outcomes were nurses’ intention to leave their current position, planned reduction in clinical hours, and pursuit of travel nursing, all within the year. The 2023 respondents who reported plans to leave were asked to indicate their next career step (eg, leave current employer but remain in nursing, retire or stop working, remain with current employer but change nursing position, leave nursing profession for another career, or pursue additional education) and the primary reason for their planned departure (eg, workloads, management and leadership concerns, pay and benefits, family and/or caregiving responsibilities, workplace abuse or violence, or employer’s approach to the COVID-19 pandemic).

Identical workplace assessment questions were asked in the 2022 and 2023 surveys, except the neutral rating was omitted from the 2023 survey. Emotional exhaustion was measured using the Oldenburg Burnout Inventory (score range: 1-5, with higher scores indicating more emotional exhaustion).^11^ Participants rated their agreement with 8 items using a 4-point Likert scale, ranging from strongly agree to strongly disagree. Participants with mean values across all items that exceed 2.25 have a clinically notable degree of burnout.^12^ In the 2022 survey, participants rated their job satisfaction using an established 5-point scale, ranging from extremely satisfied to extremely dissatisfied.^13^ In the 2023 survey, a 4-point scale was used to harmonize participants’ answers with those in a simultaneous physician survey (which will be reported separately). Nurses rated their practice environment’s delivery of high-quality care as favorable, mixed, or unfavorable.^14^ Nurses reported workplace abuse or violence in the past year (physical, verbal, sexual, or bullying, collapsed into 1 category).^15^ Additionally, nurses reported their staffing levels in the past week worked (overstaffed, staffed appropriately, or understaffed) and use of mandatory overtime (not used, used rarely, or used frequently).^8^

Participant characteristics were obtained similarly in the 2022 and 2023 surveys. Factors included age (categorical), advanced practice RN (APRN) role (yes or no), primary practice setting (as categorized by the annual Survey of Michigan Nurses^16^), gender identity, and race and ethnicity. Given that the US nursing population is composed of predominantly White and female individuals, exploring the differences across these factors could inform workforce diversity considerations.

### Statistical Analysis

In this study, χ^2^ statistics were used to compare differences in employment plans and workplace assessments between the 2022 and 2023 survey cohorts. Multivariable logistic regression was used to evaluate the likelihood of 2023 survey respondents leaving their current employer within 12 months. Given the number of personal and workplace factors identified in prior work^8^ and consistent with the holistic conceptual model posited by the National Academy of Medicine Action Collaborative,^10^ we used backward selection with a threshold of α = .05 to identify a parsimonious set of variables for inclusion in the reported logistic regression model. Statistical significance for all analyses was preestablished at 2-sided *P* = .05. Data analysis was performed with SAS 9.4 (SAS Institute Inc).

The study protocol allowed participants to skip any survey questions they wished. Therefore, a small amount of missing data was present across all variables and outcomes. For descriptive analyses, missing values were treated as a distinct category. For regression analyses, complete case analysis was used.

Three sensitivity analyses were performed, and all were directed toward the multivariable analysis. First, to address the potential implications of missing data, we estimated a logistic regression model with multiple imputation as opposed to the complete case analysis. Second, in addition to backward selection, we performed forward and best set analysis, which generated similar results. Third, we estimated a regression model on the subset of respondents who were APRNs. Advanced practice nurses have unique practice patterns and employment relationships; hence, it was important to examine them separately for potential differences. The results with the imputation model and a model restricted solely to APRNs are summarized in eTables 1 and 2 in Supplement 1.

## Results

In 2022, 13 687 eligible nurses responded to the survey for an overall response rate of 8.3%.^17^ The 2023 survey participants included 10 277 nurses for a response rate of 7.4% (eFigure in Supplement 1). This study focused on RNs in active practice, totaling 9150 in 2022 (6495 females [71.0%], 736 males [8.0%], and 62 other gender identity [0.7%]; 136 Asian [1.5%], 270 Black [3.0%], 111 Hispanic or Latino [1.2%], 33 Native American, Alaska Native, Native Hawaiian, or Pacific Islander [0.4%], 6284 White [68.7%], and 189 multiple [2.1%] races and ethnicities) and 7059 in 2023 (5134 females [72.7%], 534 males [7.6%], 19 other gender identity [0.3%]; 117 Asian [1.7%], 219 Black [3.1%], 74 Hispanic or Latino [1.1%], 23 Native American, Alaska Native, Native Hawaiian, or Pacific Islander [0.3%], 4945 White [70.1%], and 165 multiple [2.3%] races and ethnicities). Table 1 shows the characteristics of the nurse participants included in the current analysis. Compared with the annual Survey of Michigan Nurses cohort,^16^ both 2022 and 2023 samples of the Michigan Nurses’ Study included relatively lower proportions of nurses aged 65 years or older and relatively higher proportions of nurses with APRN degrees. Demographics of 2022 and 2023 survey respondents were comparable.

**Table 1.** Participant Characteristics

Characteristic	Participants, No. (%)			
	Survey of Michigan Nurses^a^		Michigan Nurses’ Study	
	2022 (n = 169 808)	2023 (n = 173 419)	2022 (n = 9150)	2023 (n = 7059)
Age, y				
≤34	38 582 (22.7)	40 109 (23.1)	1385 (15.1)	912 (12.9)
35-44	38 819 (22.8)	40 911 (23.6)	1611 (17.6)	1202 (17.0)
45-54	35 572 (20.9)	36 285 (20.9)	1838 (20.1)	1344 (19.0)
55-64	33 675 (19.8)	33 082 (19.1)	1914 (20.9)	1646 (23.3)
≥65	23 112 (13.6)	22 993 (13.2)	568 (6.2)	678 (9.6)
Missing data or unknown	48 (0.2)	39 (0.1)	1834 (20.0)	1277 (18.1)
APRN^b^				
Yes	16 765 (9.9)	17 857 (10.3)	1021 (11.2)	865 (12.3)
No	153 043 (90.1)	155 562 (89.7)	6289 (68.7)	4928 (69.8)
Missing data or unknown	NA	NA	1840 (20.1)	1266 (17.9)
Gender identity				
Female	NA	NA	6495 (71.0)	5134 (72.7)
Male			736 (8.0)	534 (7.6)
Another choice^c^			62 (0.7)	19 (0.3)
Missing data or unknown			1857 (20.3)	1372 (19.4)
Race and ethnicity^d^				
Asian	NA	NA	136 (1.5)	117 (1.7)
Black			270 (3.0)	219 (3.1)
Hispanic or Latino			111 (1.2)	74 (1.1)
Native American, Alaska Native, Native Hawaiian, or Pacific Islander			33 (0.4)	23 (0.3)
White			6284 (68.7)	4945 (70.1)
Multiple responses			189 (2.1)	165 (2.3)
Write-in answer			185 (2.0)	144 (1.9)
Missing data or unknown			1942 (21.2)	1372 (19.4)
Primary practice setting				
Community or public health	NA	NA	746 (8.2)	665 (9.4)
Nursing education			184 (2.0)	133 (1.9)
Inpatient or acute care			3887 (42.5)	2648 (37.5)
Long-term care			331 (3.6)	319 (4.5)
School nurse			112 (1.2)	105 (1.5)
Other setting			2074 (22.7)	1936 (27.4)
Missing data			1816 (19.9)	1253 (17.8)

Abbreviations: APRN, advanced practice registered nurse; NA, not applicable.

^a^ The annual survey of Michigan nurses is conducted on behalf of the state based on licensure data and captures basic categorical information on age and whether the nurse holds an advanced practice certification.

^b^ Includes nurse practitioners, nurse anesthetists, midwives, and clinical nurse specialists.

^c^ Information suppressed to maintain participant privacy. Choices included female, male, transgender, nonbinary, gender nonconforming, or prefer another choice.

^d^ Identified by participants, who could choose from multiple race and ethnicity categories.

### Employment Plans Among Surveyed Nurses

**Figure 1.**
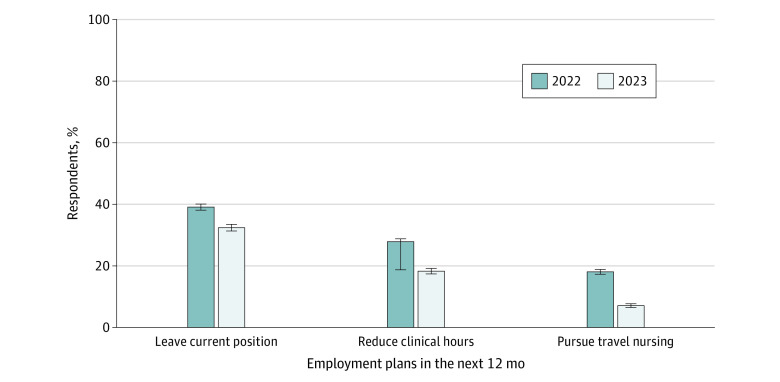
Practicing Nurses’ Planned Job Changes in the 2022 and 2023 Surveys Differences between 2022 and 2023 responses were significant at *P* < .05. Error bars represent 95% CIs.

Figure 1 compares participants’ plans for employment for the 2022 and 2023 samples. In 2022, 3576 nurses (39.1%) surveyed planned to leave their position within the next year, 2549 (27.9%) planned to reduce their clinical hours, and 1652 (18.1%) planned to pursue travel nursing. In 2023, these 3 outcomes were less frequently reported, with 2259 nurses (32.0%) planning to leave their position, 1270 (18.0%) planning to reduce clinical hours, and 494 (7.0%) planning to pursue travel nursing (*P* < .05).

**Figure 2.**
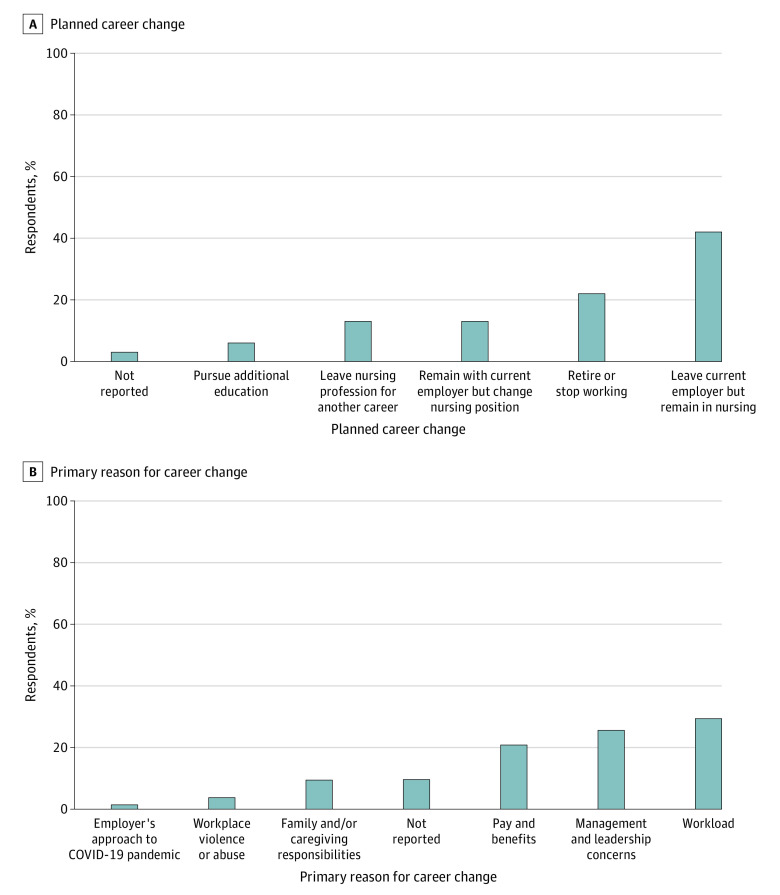
Nurse-Reported Career Change Plans and Reasons in the 2023 Survey There were 2287 survey respondents in 2023.

Among 2287 respondents to the 2023 survey who planned on leaving within the year, 957 (41.8%) planned to leave their current employer but remain in nursing, 514 (22.5%) planned to retire or stop working, 307 (13.4%) planned to remain with current employer but change nursing position, 299 (13.1%) planned to leave the nursing profession for another career, and 139 (6.1%) planned to pursue additional education (Figure 2A). When combining those planning to seek a non-nursing position and those planning to retire, we found a total of 813 RNs (35.5%) with plans to exit the field of nursing. Among these respondents, the primary reasons for departure were workloads (672 [29.4%]), management and leadership concerns (585 [25.6%]), and pay and benefits (476 [20.8%]) (Figure 2B).

### Differences in Working Conditions Between 2022 and 2023 Cohorts

Notable differences in workplace assessments were reported between the 2022 and 2023 surveys (Table 2). The mean (SD) nurse emotional exhaustion score was significantly lower for 2023 respondents than for the 2022 sample (2.96 [0.62] vs 3.09 [0.44]; *P* < .001). The proportion of nurses who were either dissatisfied or extremely dissatisfied in their current position was similar for both 2022 and 2023 cohorts (26.5% and 28.2%). Fewer nurses in the 2023 cohort rated their practice environment as unfavorable compared with nurses in the 2022 cohort (791 [11.2%] vs 1542 [16.9%]; *P* < .001), with a similar pattern observed for reported workplace abuse or violence in the past year (3063 [43.4%] vs 4591 [50.2%]; *P* < .001). While over 40% of nurse respondents in both years reported understaffing in their past week worked, fewer nurses reported this concern in the 2023 survey (2898 [41.0%] vs 4407 [48.2%]; *P* < .001). A higher proportion of nurses in the 2023 survey reported that mandatory overtime was not used in their workplace (47.3% vs 37.6%; *P* < .001). Reported use of frequent mandatory overtime decreased across respondents (1709 [18.7%] in 2022 to 824 [11.7%] in 2023; *P* < .001).

**Table 2.** Differences in Workplace Assessments Between 2022 and 2023 Surveys

Measure	Participants, No. (%)		*P* value
	2022 (n = 9150)	2023 (n = 7059)	
Emotional exhaustion score, mean (SD)^a^	3.09 (0.44)	2.96 (0.62)	<.001
Job satisfaction			
Extremely satisfied	1115 (12.2)	1660 (23.5)	NA^b^
Satisfied	3431 (37.5)	3249 (46.0)	
Neutral	1952 (21.3)	NA^b^	
Dissatisfied	1885 (20.6)	1484 (21.0)	
Extremely dissatisfied	541 (5.9)	506 (7.2)	
Missing data	226 (2.5)	160 (2.3)	
Current practice environment			
Favorable	2566 (28.0)	2732 (38.7)	<.001
Mixed	4803 (52.5)	3365 (47.7)	
Unfavorable	1542 (16.9)	791 (11.2)	
Missing data	239 (2.6)	171 (2.4)	
Workplace abuse or violence in past 12 mo			
No	3392 (37.1)	3482 (49.3)	<.001
Yes	4591 (50.2)	3063 (43.4)	
Missing data	1167 (12.7)	514 (7.3)	
Staffing in past wk worked			
Overstaffed	102 (1.1)	69 (1.0)	<.001
Staffed appropriately	2959 (32.3)	2751 (39.0)	
Understaffed	4407 (48.2)	2898 (41.0)	
Missing data	1682 (18.4)	1341 (19.0)	
Mandatory overtime use			
Not used	3439 (37.6)	3337 (47.3)	<.001
Used rarely	1978 (21.6)	1502 (21.3)	
Used frequently	1709 (18.7)	824 (11.7)	
Missing data	2024 (22.1)	1396 (19.8)	

Abbreviation: NA, not applicable.

^a^ As measured by Oldenburg Burnout Inventory (score range: 1-5, with higher scores indicating more emotional exhaustion).

^b^ A neutral option was not offered in the 2023 survey. Given the difference in question options, a χ^2^ analysis was not performed to compare 2022 and 2023 responses for this question.

### Factors Associated With Plans to Leave the Nursing Profession

The sample size for this analysis was restricted to 5445 participants who were practicing as an RN in 2023 and had requisite data for analysis (Table 3). Among the 2023 survey respondents, factors associated with an increased likelihood of planning to leave the nursing profession were an abusive or violent workplace event in the past 12 months (odds ratio [OR], 1.39; 95% CI, 1.05-1.82) and higher emotional exhaustion scores (OR, 3.05; 95% CI, 2.38-3.91). Compared with those who reported unfavorable work environments, nurses who rated their environment as favorable (OR, 0.37; 95% CI, 0.22-0.62) or mixed (OR, 0.67; 95% CI, 0.49-0.90) were significantly less likely to plan to leave nursing. Nurses who reported excellent (OR, 0.28; 95% CI, 0.14-0.56), good (OR, 0.34; 95% CI, 0.20-0.59), or acceptable (OR, 0.48; 95% CI, 0.29-0.80) clinical setting safety were also significantly less likely to have such plans. While younger vs older nurses were more likely to plan to leave their current position, these differences were not significant in the complete case analysis.

**Table 3.** Estimated Association Between Selected Factors and Plan to Leave the Nursing Profession Within 12 Months in the 2023 Survey^a^

Variable	OR (95% CI)	*P* value
Abusive or violent workplace event in past 12 mo	1.39 (1.05-1.82)	.02
Current practice environment		
Favorable	0.37 (0.22-0.62)	<.001
Mixed	0.67 (0.49-0.90)	.008
Unfavorable	1 [Reference]	
Emotional exhaustion score^b^	3.05 (2.38-3.91)	<.001
Current clinical setting safety rating		
Excellent	0.28 (0.14-0.56)	<.001
Good	0.34 (0.20-0.59)	<.001
Acceptable	0.48 (0.29-0.80)	.005
Poor	0.69 (0.42-1.12)	.13
Terrible	1 [Reference]	
Participant age, y		
≤34	1.17 (0.83-1.64)	.38
35-44	1.36 (1.00-1.87)	.05
45-54	1 [Reference]	
55-64	0.80 (0.57-1.14)	.22
≥65	0.76 (0.42-1.38)	.37

Abbreviation: OR, odds ratio.

^a^ There were 5445 survey respondents in 2023.

^b^ As measured by Oldenburg Burnout Inventory (score range: 1-5, with higher scores indicating more emotional exhaustion).

In a sensitivity analysis in which a multiple imputation model was used to account for missing data, nurses between 35 and 44 years of age were significantly more likely to report plans to leave than nurses between 45 and 55 years of age (OR, 1.41; 95% CI, 1.04-1.90) (eTable 1 in Supplement 1). Otherwise, these findings were consistent with primary results.

In another sensitivity analysis restricted to nurses who reported being APRNs, similar to our model with all nurses, APRNs were significantly more likely to plan to leave nursing as a profession if they reported higher emotional exhaustion scores (OR, 2.21; 95% CI, 1.16-4.20) (eTable 2 in Supplement 1). These APRNs were also significantly less likely to have such plans if they deemed their work environment as favorable (OR, 0.15; 95% CI, 0.04-0.55) and the quality of care provided in their primary work setting as excellent (OR, 0.17; 95% CI, 0.04-0.80).

## Discussion

This survey study showed persistently high planned departures from the RN workforce in Michigan, the tenth most populous US state. Unfavorable workplace assessments decreased in the 2023 cohort compared with the 2022 sample. Specifically, fewer nurses in the 2023 survey reported plans to leave their current position, reduce their clinical hours, or pursue travel nursing. These findings suggest somewhat improved working conditions and possible easing of workforce pressures. Based on these findings, additional work is needed to elucidate why and how conditions are improving. As an example, the reduction in mandatory overtime use over the 2 time points suggests that hospital executives recognized the adverse consequences of this managerial practice and have adjusted related policies. Analyses by specific clinical settings would add granularity to the findings and potentially target intervention strategies more precisely.

While improvements have been noted, lingering concerns persist. Over one-third of practicing nurses surveyed in 2023 indicated a plan to leave their current position, suggesting a higher potential for a destabilized health care workforce. Nurses were more likely to plan to leave their employer if they had experienced an abusive or violent workplace event, reported higher levels of emotional exhaustion, and rated their hospital more poorly on patient safety. Employer’s COVID-19 response was not cited frequently as a primary reason for departure. Both the 2022 and 2023 surveys were conducted in early spring into summer, when COVID-19 cases in Michigan were not at peak incidence, further discounting the COVID-19 pandemic approach as a factor in job dissatisfaction.^18^

Placed into context, the findings from these 2 surveys conducted approximately 1 year apart indicate moderately improved but persistently problematic workplace environments for RNs. Michigan nurses reported high emotional exhaustion or burnout scores, understaffed patient care areas, occurrence of abusive or violent workplace events in the past 12 months, and job dissatisfaction. In a study of nurses from New York and Illinois who left their employers between 2018 and 2021, planned retirement, burnout, and insufficient staffing were the most frequently cited reasons for departure.^19^ This analysis also identified a worrisome pattern, which was reported in a prior study,^8^ of high rates of job dissatisfaction and intention to leave their position among nurses aged 34 years or younger. This finding, now observed in at least 2 large surveys, calls into question whether gains in the RN workforce will be sufficient to offset these planned departures.

There may be larger societal factors associated with the high rate of planned departures observed in the present study, including general economic conditions, family factors, and alternative job opportunities. A 2023 cross-sector survey of job satisfaction revealed that only half of US individuals are satisfied at work,^20^ with the lower rates of satisfaction observed among employees younger than 50 years. Yet compared with staff in other employment sectors, health care workers are more difficult to train and orient due to their specialized knowledge and skills. This finding suggests that novel retention strategies are needed for this unique sector of the workforce.

An underappreciated but concerning finding is that between 18.0% (in 2023) and 27.9% (in 2022) of respondents indicated plans to reduce their clinical hours. Health care systems rely on a large part-time nursing workforce to fill acute and persistent staffing gaps. A high proportion of nurses planning to reduce their clinical hours suggests that fewer nurses will be available to serve this vital function, a situation which may be associated with increased demand for potentially expensive temporary personnel.

The study provides insights into the future plans and motivations of dissatisfied nurses, most of whom intend to remain in nursing but wish to leave their current employers, affirming the adage that many nurses love their work but hate their jobs.^21^ Dissatisfied nurses consistently identified workloads as a primary reason for their dissatisfaction. Strategies should focus on retaining practicing nurses, and the priority tactic recommended by nurses is to increase nurse staffing levels above the current norms. There are multiple ways to improve nurse staffing, including administratively within systems and through policy changes.^22^ A second concern for dissatisfied nurses involved management and leadership, which could be addressed through proactive listening and trust-building efforts.

It is not surprising that workplace assessments were similar in both survey years, as hospital executives and policymakers have enacted few changes to improve nursing practice environments in tangible ways. However, policy strategies are available both for retention of practicing nurses and recruitment of individuals to nursing.^23^ At the state level, a bipartisan package of legislation that addressed hospital staffing levels and changes to the Board of Nursing to facilitate licensure approvals in Michigan was introduced but not enacted. On December 6, 2023, after both surveys were completed, Michigan Governor Gretchen Whitmer signed legislation that steepens penalties for assaulting health care workers and requires health care facilities to post corresponding signage.^24^ The impact of this legislation has yet to be evaluated.

Federal initiatives to increase staffing and decrease workplace abuse or violence have been proposed without legislative action. In July 2023, the American Nurses Association endorsed enforceable, minimum nurse staffing levels in hospitals, citing concerns for patient safety and nurse well-being.^25^ In August 2023 (after the second survey was closed), the US Health Resources and Services Administration announced a $100 million package that supports licensed practical nurses to complete RN programs and growth of the pool of APRNs and nursing school faculty.^26^ It is unclear how this substantial financial boost to the supply of nurses in various roles will address the key concern identified in this study: retention of practicing and experienced RNs within health care systems.

### Study Limitations

Several limitations of this study merit consideration. The low survey response rates for a statewide sample resemble similarly large surveys of health care professionals. While we did not observe stark demographic differences between responders and nonresponders, the sample sizes may not be representative of the RN population, specifically nonresponders satisfied with their positions. Due to the study design and absence of identifiers, there is likely a substantial overlap between 2022 and 2023 responders. The design does not permit the identification of temporal or causal associations between job outcomes and workplace or personal factors. Given concerns that many nonresponders may have already left health care employment (and thus chose not to complete the survey), we did not focus this analysis on those who had already ended their employment. Relatedly, we were unable to track participants’ actual job changes.

## Conclusions

The 2023 respondents to the Michigan Nurses’ Study reported improved workplace assessments; however, planned departures, abusive or violent events, and unsafe conditions continue to permeate nurses’ workplaces, putting patients at risk for adverse events and nurses at risk for burnout. Few policy provisions have been enacted to address the primary reason for planned departures: persistent understaffing. Health care professionals and the public at large can query elected officials and candidates for office on their proposals to retain practicing RNs and expand the population of nurses available to meet the growing need for high-quality, equitable health care. Health system leaders and policymakers should prioritize initiatives that support nurse retention and reduce potential workforce instability.

## Notes

Supplement 1. **eFigure.** CONSORT Diagram for 2023 Nurse Participants **eTable 1.** Odds Ratios Estimating the Association Between Selected Factors and Plan to Leave the Nursing Profession Within the Next Twelve Months, Using Multiple Imputation. N=7,059 **eTable 2.** Odds Ratios Estimating the Association Between Selected Factors and Plan to Leave the Nursing Profession Within the Next Twelve Months, Restricted to Advanced Practice Nurses. N=786

Supplement 2.
Data Sharing Statement
