# Supplementary material for: Changes in Registered Nurse Employment Plans and Workplace Assessments
Source: JAMA Netw Open. 2024 Jul 18;7(7):e2421680. doi: 10.1001/jamanetworkopen.2024.21680 (PMC11258586; doi:10.1001/jamanetworkopen.2024.21680)
Supplement: Supplement 1. — eFigure. CONSORT Diagram for 2023 Nurse Participants eTable 1. Odds Ratios Estimating the Association Between Selected Factors and Plan to Leave the Nursing Profession Within the Next Twelve Months, Using Multiple Imputation. N=7,059 eTable 2. Odds Ratios Estimating the Association Between Selected Factors and Plan to Leave the Nursing Profession Within the Next Twelve Months, Restricted to Advanced Practice Nurses. N=786 [file jamanetwopen-e2421680-s001.pdf]

Supplemental Online Content

Friese CR, Medvec BR, Marriott DJ, et al. Changes in registered nurse employment plans and workplace assessments. *JAMA Netw Open*. 2024;7(7):e2421680. doi:10.1001/jamanetworkopen.2024.21680

eFigure. CONSORT Diagram for 2023 Nurse Participants

eTable 1. Odds Ratios Estimating the Association Between Selected Factors and Plan to Leave the Nursing Profession Within the Next Twelve Months, Using Multiple Imputation. N=7,059

eTable 2. Odds Ratios Estimating the Association Between Selected Factors and Plan to Leave the Nursing Profession Within the Next Twelve Months, Restricted to Advanced Practice Nurses. N=786

This supplemental material has been provided by the authors to give readers additional information about their work.

eFigure. CONSORT Diagram for 2023 Nurse Participants

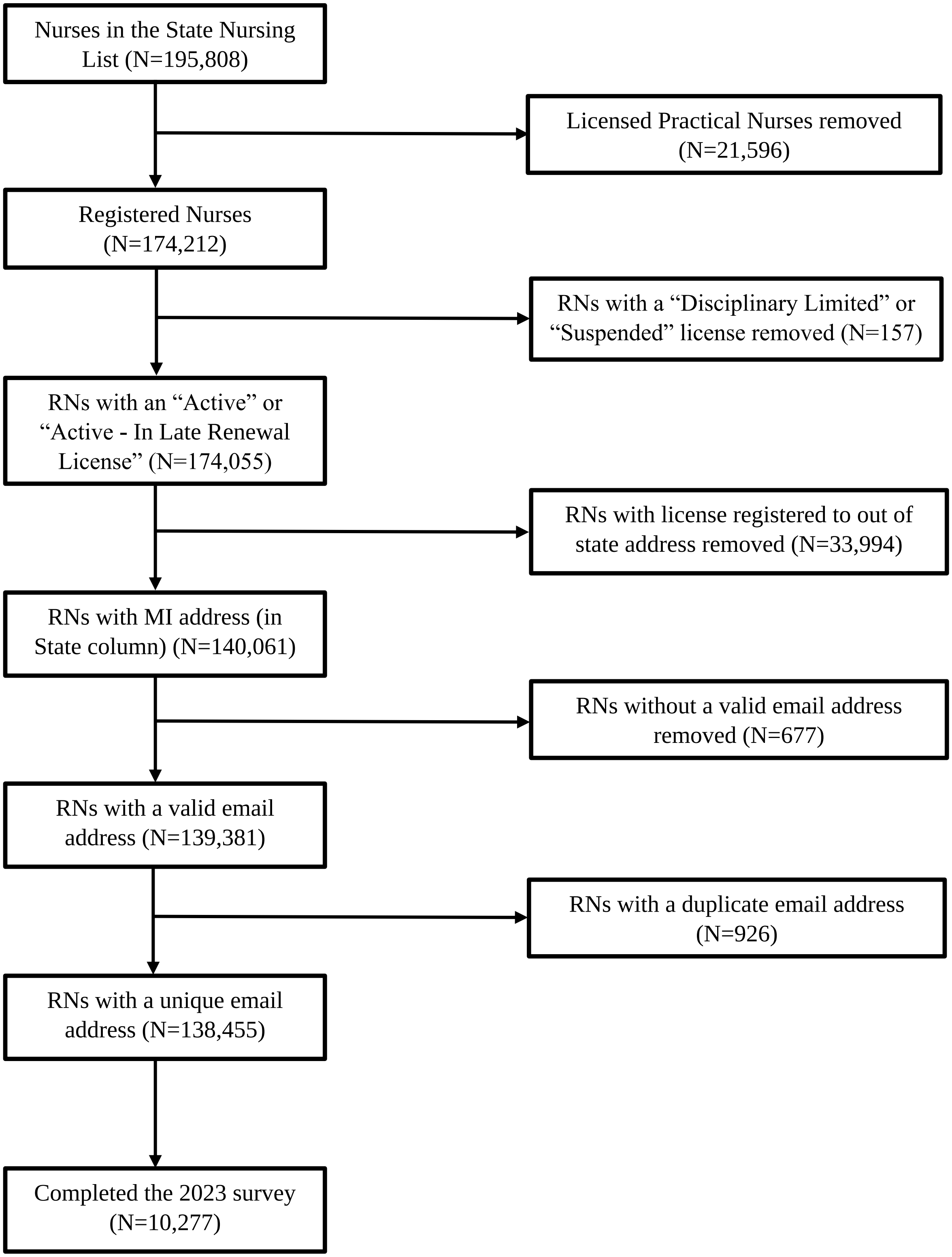

© 2024 Friese CR et al. *JAMA Network Open*

eTable 1. Odds Ratios Estimating the Association between Selected Factors and Plan to Leave the Nursing Profession within the next Twelve Months, using Multiple Imputation. N=7,059

Variable	Odds Ratio	(95% CI)	*P* Value
Workplace abusive event in past twelve months	1.39	(1.05-1.82)	.02
Current practice environment			
• Favorable environment	0.35	(0.22-0.55)	<.001
• Mixed environment	0.67	(0.51-0.88)	<.01
• Unfavorable environment	Reference	Reference	
Oldenburg Exhaustion Scale	2.91	(2.33-3.64)	<.001
Current practice safety rating			
• Excellent	0.42	0.27-0.67	<.001
• Good	0.25	0.14-0.47	<.001
• Acceptable	0.29	0.17-0.47	<.01
• Poor	0.65	0.41-1.01	0.06
• Terrible	Reference	Reference	
Participant Age			
• 34 and under	1.23	0.90-1.69	0.20
• 35-44	1.41	1.04-1.90	0.02
• 45-54	Reference	Reference	
• 55-64	0.80	0.57-1.11	0.18
• 65 and over	0.74	0.43-1.25	0.26

OR: Odds Ratio; 95% CI: 95% confidence interval

© 2024 Friese CR et al. *JAMA Network Open*

eTable 2. Odds Ratios Estimating the Association between Selected Factors and Plan to Leave the Nursing Profession within the next Twelve Months, restricted to Advanced Practice Nurses. N=786

Variable	Odds Ratio	(95% CI)	*P* Value
Current practice environment			
• Favorable environment	0.15	(0.04, 0.55)	<.01
• Mixed environment	0.32	(0.15, 0.70)	<.01
• Unfavorable environment	Reference	Reference	
Oldenburg Exhaustion Scale	2.21	(1.16, 4.20)	0.02
Quality of care in primary setting			
• Excellent	0.17	(0.04, 0.80)	0.03
• Good	0.19	(0.06, 0.61)	<0.01
• Fair	0.30	(0.10, 0.87)	0.03
• Poor	Reference	Reference	

© 2024 Friese CR et al. *JAMA Network Open*
