# Supplementary material for: Changes in Registered Nurse Employment Plans and Workplace Assessments
Source: JAMA Netw Open. 2024 Jul 18;7(7):e2421680. doi: 10.1001/jamanetworkopen.2024.21680 (PMC11258586; doi:10.1001/jamanetworkopen.2024.21680)
Supplement: Supplement 2. — Data Sharing Statement [file jamanetwopen-e2421680-s002.pdf]

Data Sharing Statement

Friese. Changes in Registered Nurse Employment Plans and Workplace Assessments. *JAMA Netw Open*. Published July 18, 2024. doi:10.1001/jamanetworkopen.2024.21680

Data
Data available: No

Additional Information
Explanation for why data not available: the sensitive nature of the data and existing data use agreements do not permit widespread availability. Interested investigators can contact the corresponding author to discuss options.
